# Role of Oxidative Stress and Apoptosis in the Placental Pathology of *Plasmodium berghei* Infected Mice

**DOI:** 10.1371/journal.pone.0032694

**Published:** 2012-03-01

**Authors:** Lalita Sharma, Jagdeep Kaur, Geeta Shukla

**Affiliations:** 1 Department of Microbiology, Panjab University, Chandigarh, India; 2 Department of Biotechnology, Panjab University, Chandigarh, India; University of Copenhagen, Denmark

## Abstract

Placental malaria is a common clinical complication during pregnancy and is associated with abortion, premature delivery, intrauterine growth retardation and low birth weight. The present study was designed to delineate the underlying mechanism of placental pathology during malarial infection with special reference to oxidative stress and apoptosis. Experimentally, pregnant BALB/c mice were infected with *Plasmodium berghei* infected red blood cells on gestation day 10. The presence of malarial infection in placenta was confirmed by histopathological studies. It was observation that infected placenta had plugged placental sinusoids with parasitized red blood cells and malarial pigments. Interestingly, we found significant increase in the level of malondialdehyde, the index of oxidative stress and decreased activity of catalase, the antioxidant in infected placenta. Furthermore, in infected placenta the oxidative stress mediated apoptosis was determined by DNA fragmentation assay, ethidium bromide/acridine orange staining and caspase activity. It was observed that oxidative stress begin after second day of malarial infection. Interestingly, it was observed that there was down regulation of anti-apoptotic protein Bcl-2 and up regulation of pro-apoptotic protein Bax in infected placenta, suggesting the involvement of mitochondrial pathway of apoptosis which was further confirmed by activation of caspase 9. However, no change in the expression of Fas gene and caspase 8 activity, indicated the absence of death receptor pathway. Thus, it can be concluded that the placental pathology during malarial infection is mediated by mitochondrial pathway of apoptosis occurring due to augmented lipid peroxidation which may in turn jeopardise the materno-fetal relationship.

## Introduction

Malaria causes about 2.7 million deaths annually worldwide, most of which are in children and pregnant women [1]. Despite all the efforts made to control malaria among pregnant women in endemic areas, malaria remains a significant cause of maternal and infant mortality and morbidity. In sub-Saharan Africa, the World hardest hit area by malaria, the infection is estimated to cause 4,00,000 cases of severe maternal anaemia and about 2,00,000 infant deaths [2], [3], [4].

Malarial infection particularly caused by *Plasmodium falciparum* is more severe and frequent in pregnant women than in non-pregnant women. It is primigravidae who are more susceptible to the infection than multigravidae [5]. Malarial infection during pregnancy results in a wide range of adverse effects like anaemia, fever, hypoglycaemia, cerebral malaria, pulmonary oedema, puerperal sepsis, haemorrhage and even maternal death [6], [7]. Moreover, maternal malaria often leads to premature delivery, still birth, intra uterine growth retardation and low birth weight [8], [9], [10]. The symptoms and complications of malaria during pregnancy differ according to the intensity of malaria transmission in a particular area. The non- immune, primigravidae are usually the most affected in areas of high transmission where 20–40% of all babies have a low birth weight whereas, in areas of low transmission primigravidae and multigravidae are equally affected [11], [12].

Placental malaria is mainly characterized by sequestration of parasitized Red Blood Cells (pRBC), malarial pigment and infiltration of monocytes within the intervillous spaces of the placenta along with perivillous fibrinoid deposits, thickening of trophoblastic basement membrane, cytotrophoblastic proliferation, syncytiotrophoblastic damage, excessive syncytial knotting and fibrinoid necrosis [7], [13], [14]. It is believed that all these pathological alterations alter the materno-fetal exchange system and result into intra-uterine growth retardation and low birth weight [15]. Sequestration of pRBC in the placenta relies on the ability of *P. faciparum* Erythrocyte Membrane Protein 1 (PfEMP1) present on the surface of pRBC to adhere to Chondroitin Sulfate A (CSA) and Hyaluronic Acid (HA), glycosaminoglycan receptors found throughout the extracellular matrix of placenta [5]. Recently, it has been proposed that the strain of *P. falciparum* implicated in maternal malaria do not bind to CD36, a nearly universal characteristic of *P. falciparum* [16]. However, the exact mechanism underlying pathophysiology of materno-fetal organ, the placenta during malarial infection remains unexplored and warrants further study. Therefore, the present study was designed to provide a better insight into the functional impairment of the placenta during malarial infection particularly with respect to oxidative stress and apoptosis.

## Materials and Methods

### Parasite

*Plasmodium berghei* (NK 65) was employed in the study and maintained in mice by serial passage of infected blood.

### Animals

BALB/c mice, 6–8 weeks old (20–22 gm) were obtained from a colony maintained in the central animal house of Panjab University, Chandigarh, India. These animals were provided with standard pellet diet and water *ad libitum*. Care, use and disposal of animals were done in accordance with the guidelines of Panjab University Animal Ethical Committee (IAEC), Chandigarh and approved by the Committee for the Purpose of Control and Supervision on Experiments on Animals (44/99/CPCSEA). The present study was also approved by the institutional review board.

### Assessment of the first gestational day

Female mice were housed overnight with male mice of the same strain in the ratio of 2∶1 and next day early morning examined for the presence of vaginal plug. The day when vaginal plug was observed it was marked as the first gestational day (GD).

### Experimental design

The animals were divided into three groups. Group I (nonpregnant-infected, n = 6): these animals were inoculated intraperitoneally with 1×10^6^ *P. berghei* infected RBCs. Group II (pregnant, n = 18): these mice were inoculated intraperitoneally with Phosphate Buffer Saline (PBS) on GD10±2. Group III (pregnant-infected, n = 24): animals belonging to this group were inoculated with 1×10^6^ *P. berghei* infected RBC intraperitoneally on GD10±2. The GD10, a mid-pregnancy period was selected for initiation of malaria infection as in earlier studies it has been documented that infection in early pregnancy GD 6 (1^st^ trimester) resulted into 100% maternal mortality and placental pathology could not be studied [17], [18].

### Follow up of animals

Percent parasitaemia in all infected mice was monitored on every alternate day in Giemsa stained tail blood films by examining at least 500 cells. Six mice belonging to each group II and III were sacrificed by cervical dislocation on 2, 4 and 6 Post Infection (PI) and placentae were removed from live fetuses. Rest of the animals belonging to group I and III were monitored for percent parasitaemia. For isolation of RNA and staining of placental cells with ethidium bromide/acridine orange stain, placentae were processed immediately. For estimation of oxidants and antioxidants and DNA isolation placentae were stored at −20°C till further use. For histopathological studies placentae were fixed in 10% buffered formalin.

### Preparation of placental homogenates and post mitochondrial supernatant

Placental homogenates from both pregnant and pregnant-infected mice were prepared in PBS using potter Elvehjem homogenizer. For preparation of post mitochondrial supernatants, half of portions from placental homogenates were cold centrifuged at 12,000× *g* for 10 minutes and supernatants labelled as Post Mitochondrial Supernatant (PMS) Protein concentrations in placental homogenates and PMS were measured as per Lowry et al. [19] and stored at −20°C till further use.

### Lipid peroxidation assay

The amount of Malon Di Aldehyde (MDA), a measure of lipid peroxidation was quantitated according to the method of Wills [20]. In brief, 0.5 millilitre (ml) of Tris-HCl buffer (0.1 M, pH 7.4) added to 0.5 ml of placental homogenate and kept at 37°C for 2 hours. Following incubation, 1.0 ml of 10% (w/v) tri chloro acitic acid (ice-cold) added and the mixture was centrifuged at 100× *g* for 10 minutes. To 1.0 ml of supernatant, 1.0 ml of 0.67% (w/v) thiobarbituric acid added and kept in boiling water bath for 10 minutes. After cooling the tubes, 1.0 ml of distilled water was added and absorbance was measured at 532 nm. The results were expressed as nanomoles of MDA per milligram of protein, using the molar extinction coefficient of chromophore (1.56×10^5^ M^−1^ cm^−1^).

### Determination of reduced Glutathione (GSH)

The levels of GSH were estimated according to the method described by Ellman [21]. One ml of placental homogenate was precipitated with 1.0 ml of 4% sulphosalicyclic acid, kept at 4°C for at least 1 hour and centrifuged at 100× *g* for 15 minutes at 4°C. The assay mixture contained 0.1 ml of supernatant, 0.2 ml of 0.01 M Di-Thio-Nitro Benzoic acid (DTNB) and 2.7 ml of phosphate buffer (0.1 M, pH 8.0). The absorbance was measured at 412 nm and results were expressed as micromoles of GSH per milligram of protein.

### Assessment of Super Oxide Dismutase (SOD) activity

SOD (EC 1.15.1.1) activity in post mitochondrial supernatant was assayed according to the method of Kono [22]. Briefly, the reaction was initiated by addition of 0.5 ml of hydroxylamine hydrochloride to the reaction mixture containing 2.0 ml Nitro-Blue Tetrazolium (NBT) and 0.1 ml PMS. SOD activity was expressed as units of SOD per milligram of protein where one unit activity is defined as the amount of SOD required to inhibit the rate of reduction of NBT by 50%.

### Measurement of catalase activity

The catalase (EC 1.11.1.6) activity in post mitochondrial supernatant was assayed by the method of Luck [23]. To carry out the assay 100 ml of phosphate buffer (0.05 M, pH 7.2) made to which 0.16 ml of H_2_O_2_ was added. The assay mixture consisted of 3 ml of the phosphate buffer and 5 µl of PMS. Change in absorbance was recorded spectrophotometrically at 240. The results were expressed as milimoles of H_2_O_2_ decomposed per minute per milligram of proteins using the molar extinction coefficient of the chromophore (0.0394 mM^−1^ cm^−1^).

### DNA fragmentation analysis

To determine apoptosis in placenta, DNA from placenta of pregnant and pregnant-infected was isolated by the method described by Strauss [24] with minor modification. Briefly, 60–70 mg placental tissue was minced, suspended in 500 µl digestion buffer and kept at 50°C overnight. Equal volume of the Tris-saturated phenol added to the digested tissue and centrifuged at 12,000× *g* for 10 minutes. The upper layer formed after centrifugation was subjected to phenol-chloroform-isoamylalcohol extraction procedure. Finally, DNA was precipitated with chilled ethanol, washed with 70% ethanol, dried and dissolved in TRIS-EDTA buffer. Equal quantity of isolated DNA (4 µg) was electrophoresed on 1.2% agarose ethidium bromide gel and analysed by Gel Doc EZ Imager (Bio-Rad). DNA was isolated from blood of *P. berghei* infected and normal pregnant mice and resolved on agarose as control.

### Determination of apoptotic cells in placenta by ethidium bromide/acridine orange staining

Cells from placentae of both pregnant and pregnant-infected mice were isolated by teasing placentae with frosted end slides. Dispersed cell suspension was filtered using a nylon mesh and centrifuged at 100× *g* for 5 minutes at 25°C. RBC in the cell pellet were lysed with chilled 2% saponin and centrifuged at 100× *g* for 10 minutes. The sediment containing cells were washed thrice with PBS. The number of cells in the suspension were counted using haemocytometer and isolated cells were observed under fluorescence microscope after staining with ethidium bromide/acridine orange stain. Blood from infected and normal pregnant mice were also subjected to ethidium bromide/acridine orange staining.

### Reverse Transcriptase Polymerization Chain Reaction (RT-PCR) for determination of apoptosis

Placentae from both pregnant and pregnant-infected mice were used for RNA isolation using TRIzol reagent, a mixture of guanidine thiocyanate and phenol in a monophase solution (Sigma Aldrich, USA) following manufacturer's protocol. The RNA was suspended in nuclease free water and stored at −80°C. The purity of RNA was monitored in 1.5% agarose ethidium bromide gel and quantitated using the Nano-Drop 1000 spectrophotometer (Thermo Fisher Scientific Inc., UK). Equal amount of RNA (2 µg) was used for synthesis of complementary DNA (cDNA) using commercially available kit (Fermentas Life Sciences, Canada). The cDNA was stored at −20°C till further use. From the cDNA, PCR was performed using following sets of primers (provided by Genex Life Sciences Pvt. Ltd, Banglore, India): Fas (CD95) (forward primer; 5/-MAGAAGGGRAGGAGTACA-3/, reverse primer; 5/-TGCACTTGGTATTCTGGGTC-3/), Bcl-2 (forward primer; 5/-CCTGTGGATGACTGAGTACC-3/, reverse primer; 5/-GAGACAGCCAGGAGAAATCA-3/), Bax (forward primer; 5/-GTTTCATCCAGGATCGAGCAG-3/, reverse primer, 5/-CATCTTCTTCCAGATGGT-3/). β-actin, as control (forward primer; 5/-ATGGAATCCTGTGGCATCCA-3/, reverse primer, 5/-TCCACACAGAGTACTTGCGCTC-3/). PCR was performed using following PCR programme: 94°C for 2 min for initial denaturation; then 35 cycles of denaturation at 94°C for 1 min; annealing at 55°C for 1 min; extension at 72°C for 1.5 min and final elongation at 72°C for 7 min. The amplified DNA was resolved in 1.5% agarose ethidium bromide gel and analysed by Gel Doc EZ Imager (Bio-Rad).

### Determination of caspase activity

To study the activation of caspases, caspase 3, 8 and 9 activities were measured in cytosolic fraction of placenta of infected and normal mice, using commercially available kits and according to manufacturer protocol (Bio Vision, USA). Briefly, cytosol (10 µl containing 50 µg protein) was mixed in a microtiter plate with assay buffer and caspase specific substrates DEVD-pNA (para-nitro-aniline) for caspase-3, IETD-pNA for caspase-8 and LEHD- pNA for caspase-9). After 6 hours of incubation at 37°C, the absorbance of pNA released was measured at 405 nm in a microtiter plate reader. The absorbance of negative control (assay buffer and substrate) was subtracted from specific values.

### Statistical analysis

Results were expressed as mean ± standard error (SE). The inter group variation was assessed by one way analysis of variance (ANOVA) and statistical significance was calculated at P<0.05.

## Results

### Enhanced susceptibility of pregnant mice to *P. berghei* infection

**Figure 1:**
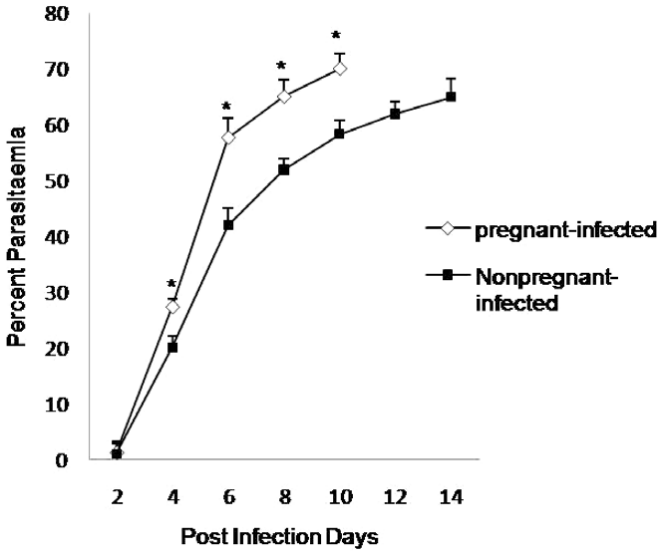
Increased percent parasitaemia in pregnant mice. BALB/c pregnant mice (GD10±2) and nonpregnant mice infected with 1×10^6^ *P. berghei* infected RBC intraperitoneally. Values are expressed as mean ± standard deviation. ^*^(p<0.05) vs. nonpregnant-infected.

Pregnant-infected and nonpregnant-infected mice had gradual increase in parasitaemic levels that began from day 2 PI. However, the percent parasitaemia in pregnant-infected mice was significantly higher (P<0.05) at each point of observation in comparison to nonpregnant mice. All pregnant-infected mice died earlier than nonpregnant-infected mice (Figure 1).

### Placental sinusoids plugged with *P. berghei* infected RBC

**Figure 2:**
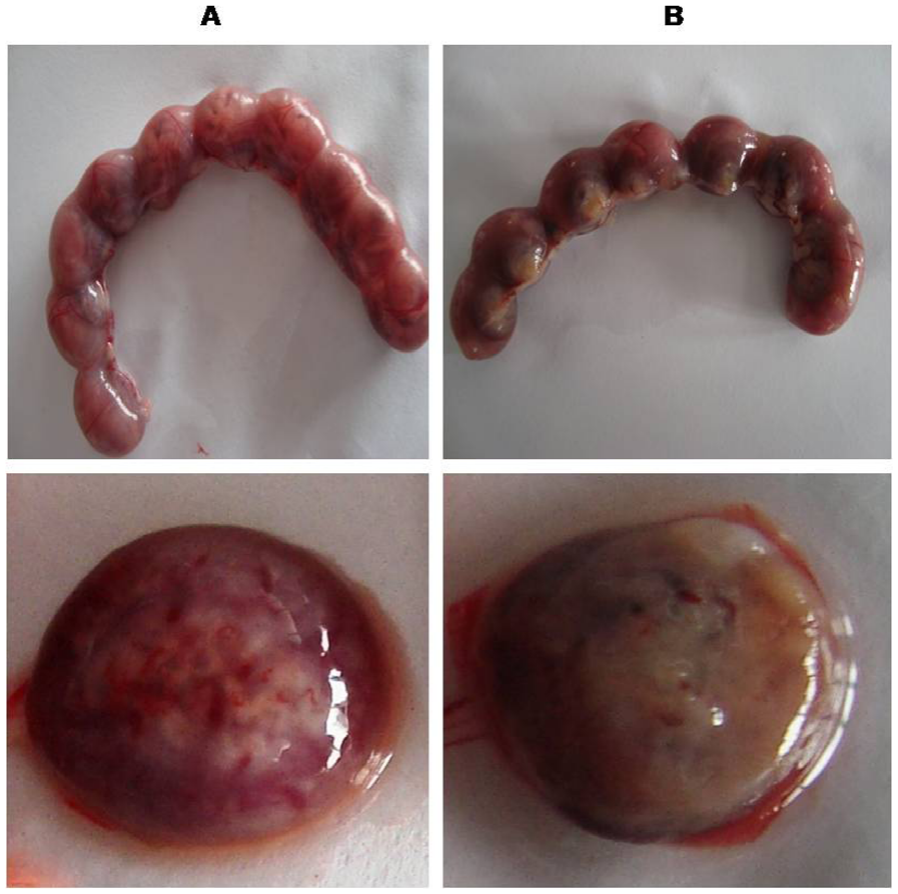
Abnormal uterus and placenta in infected mice. Panel (A): Pregnant mice showing bright red colour of the uterus and placenta. Panal (B): *P. berghei* infected mice showing dark-greyish coloured uterus and placenta.

**Figure 3:**
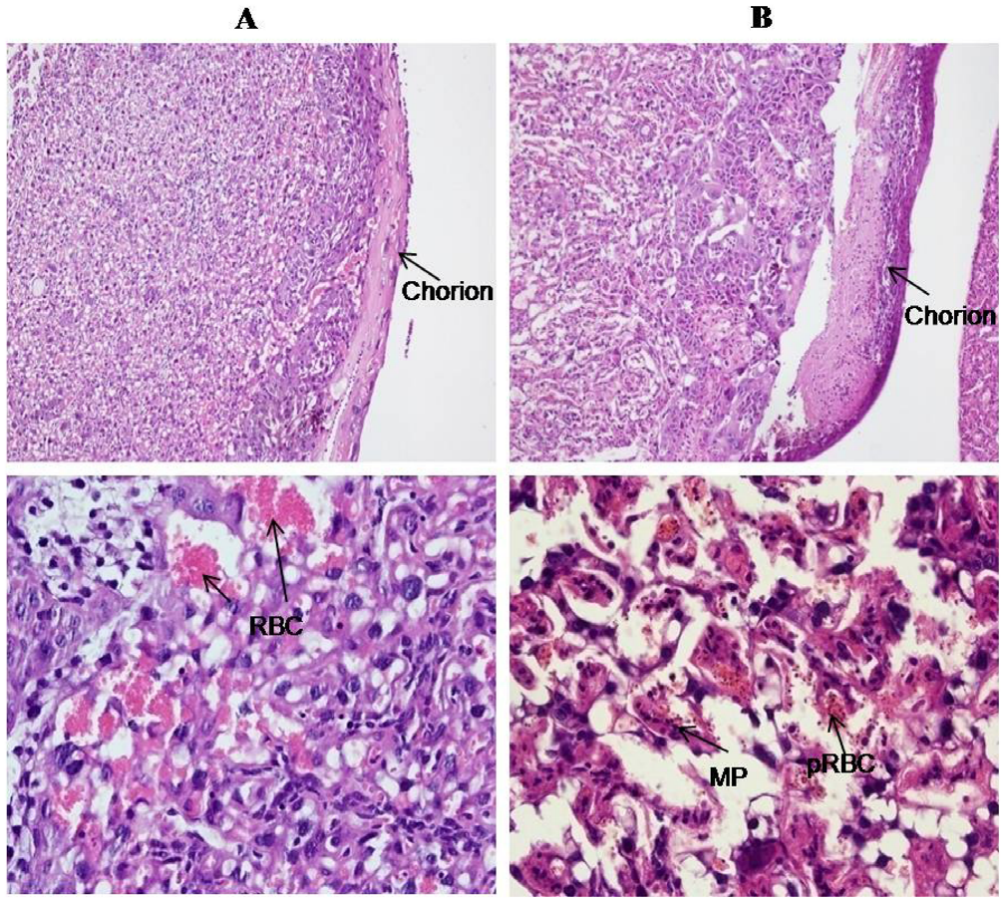
Histology of *P. bergei* infected placenta. Placentae from infected mice collected on 6 day post infection. Panel (A): Pregnant mice showing normal RBC (RBC) and normal chorion. Panel (B): *P. berghei* infected mice showing malarial pigments (MP), parasitized RBC (pRBC) and thickening of chorion. (100× & 400×, H & E staining).

The establishment of infection in placenta was confirmed both by macroscopic and microscopic observations. Macroscopically, it was observed that both the uterus and placenta of pregnant-infected mice were dark-greyish coloured, characteristics of malarial infection compared to bright red colour in pregnant mice (Figure 2). Histopathologically, the placenta showed plugging of placental sinusoids with parasitized RBCs and deposition of malarial pigment compared with the normal morphometry of placenta in pregnant mice (Figure 3).

### Increased lipid peroxidation in placenta of infected mice

**Figure 4:**
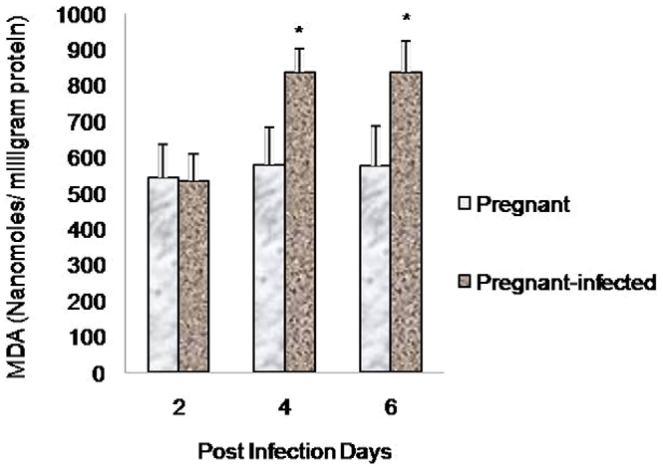
Increased lipid peroxidation in *P. berghei* infected placenta. MDA levels, an index of lipid peroxidation were measured in placenta of both pregnant and pregnant-infected mice. Values are expressed as mean ± standard deviation. ^*^(p<0.05) vs. pregnant.

The levels of MDA were measured as index of lipid peroxidation and were found to be significantly higher (p<0.05) in placenta of pregnant-infected mice compared with pregnant mice at each point of observation. However, no significant difference was observed in MDA levels in the placenta of both pregnant and pregnant-infected mice on day 2 PI (Figure 4).

### Decreased catalase activity in *P. berghei* infected placenta

**Figure 5:**
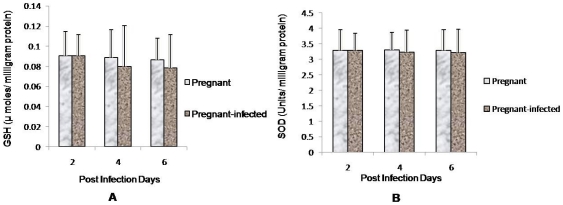
Unaltered levels of GSH and SOD in *P. berghei* infected placenta. (A, B): The levels of GSH and SOD were monitored in placenta of pregnant and pregnant-infected mice. Values are expressed as mean ± standard deviation.

**Figure 6:**
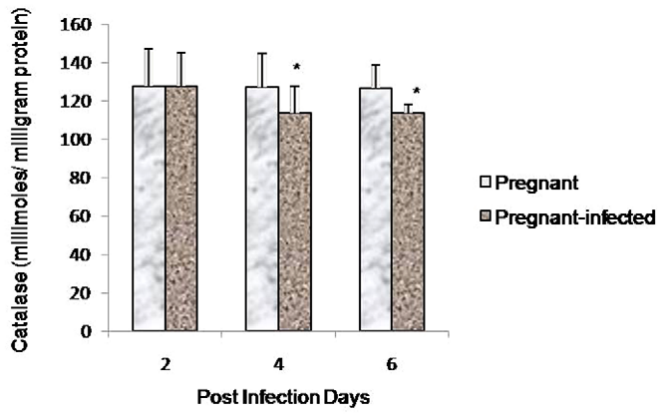
Reduced catalase activity in placenta of malaria infected mice. Catalase activity was measured in placenta of pregnant and pregnant-infected mice. Values are expressed as mean ± standard deviation. ^*^(p<0.05) vs. pregnant.

The level of GSH and SOD activity in placenta obtained either from pregnant or pregnant-infected mice remain unaltered at each point of infection (Figure 5A & 5B). However, the activity of catalase decreased significantly (p<0.05) after day 2 PI in placenta of infected mice compared with pregnant mice (Figure 6).

### DNA fragmentation in infected placenta

**Figure 7:**
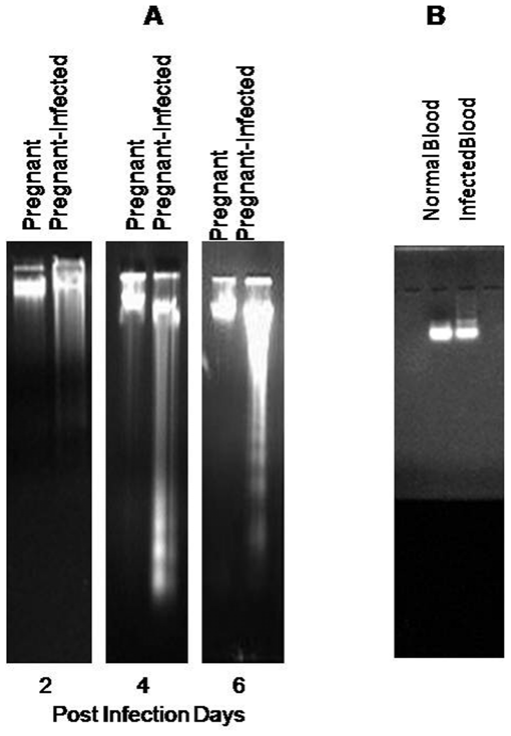
DNA fragmentation in placenta of infected mice. **A:** Placenta of *P. berghei* infected mice showing distinct DNA fragmentation on day 4 and 6 PI respectively compared with DNA of normal placenta. **B:** No DNA fragmentation observed in blood from infected and normal mouse.

As a hallmark of apoptosis, DNA fragmentation assay was performed to detect apoptosis in placenta during malarial infection. Interestingly, faint but visible DNA fragments were observed in infected placenta on day 4 and 6 PI (Figure 7A). Interestingly, blood samples obtained either from infected or normal pregnant mice did not show DNA fragmentation, indicating the absence of apoptosis (Figure 7A). Moreover, necrosis was absent in infected placental as there was no smearing of DNA (Figure 7B).

### Enhanced apoptosis in *P. berghei* infected placenta

**Figure 8:**
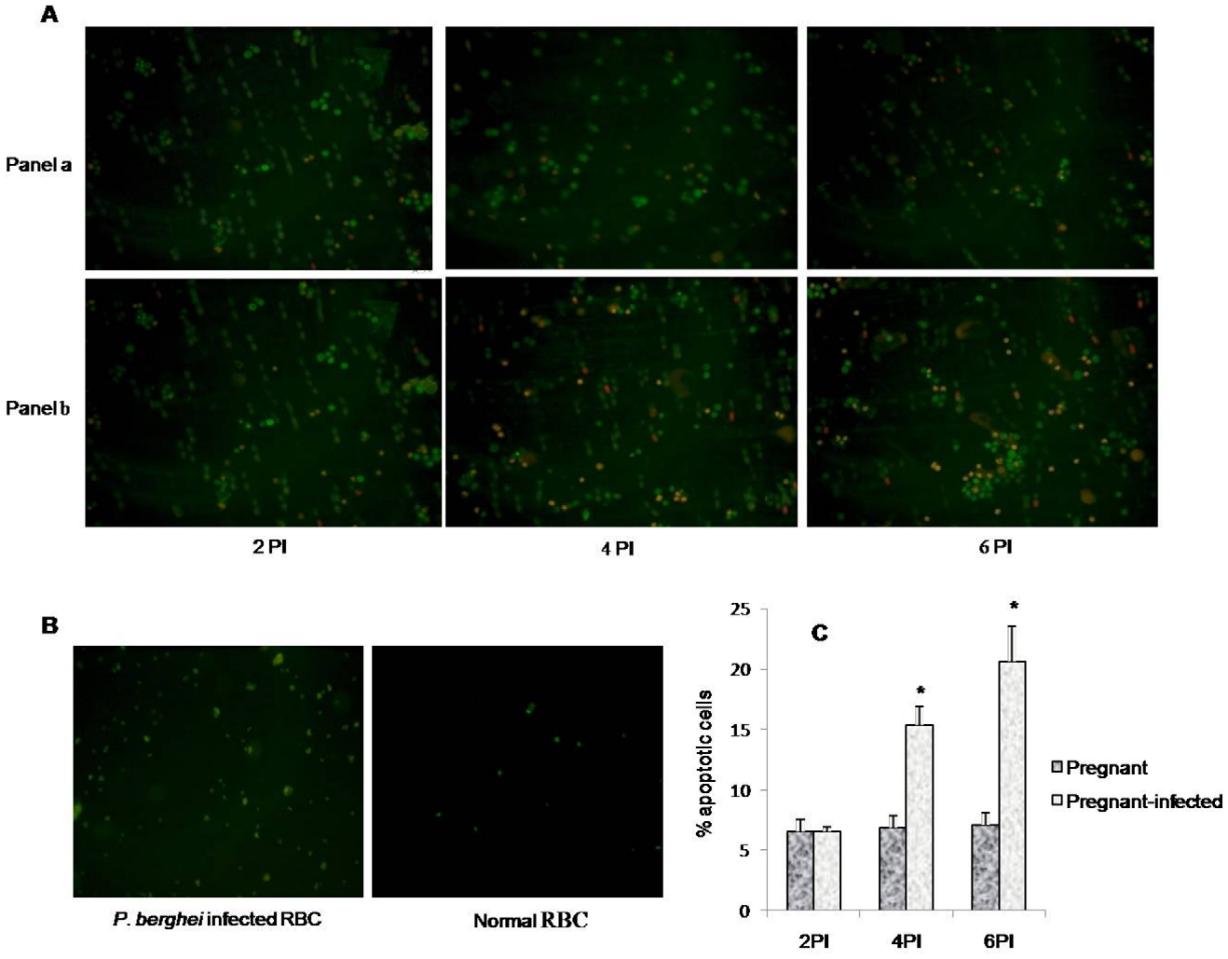
Higher apoptotic cells in ethidium bromide/acridine orange (Etbr/Ar) stained placenta of infected mice. **A:** Panel (a) showing lower number of apoptotic cells in placenta of pregnant mice (green nucleus = live cells, orange nucleus = apoptotic cells, Panel (b) showing higher number of apoptotic cells in *P. berghei* infected placenta on day 4 and 6 PI respectively. **B:** Non-apoptotic cells in control samples that are normal blood and *P. berghei* infected blood. **C:** Bar diagram represent the percentage of apoptotic cells in normal and *P. berghei* infected placenta. Values are expressed as mean ± standard deviation. ^*^(p<0.05) vs. pregnant.

Placenta of pregnant-infected mice showed significantly higher (p<0.05) number of apoptotic cells on day 4 PI and 6 PI compared to the lower number in placenta of pregnant mice as observed by ethidium bromide/acridine orange staining. It was also observed that the process of apoptosis further increased with the progression of infection (Figure 8A, 8C). Moreover, blood from infected and normal mice did not show apoptotic nuclei indicating the absence of apoptosis (Figure 8B). Therefore, majority of cells showing apoptosis in ethidium bromide/acridine orange staining are placental cells.

### Up-regulation of Bax and down-regulation of Bcl-2 expression in infected placenta

**Figure 9:**
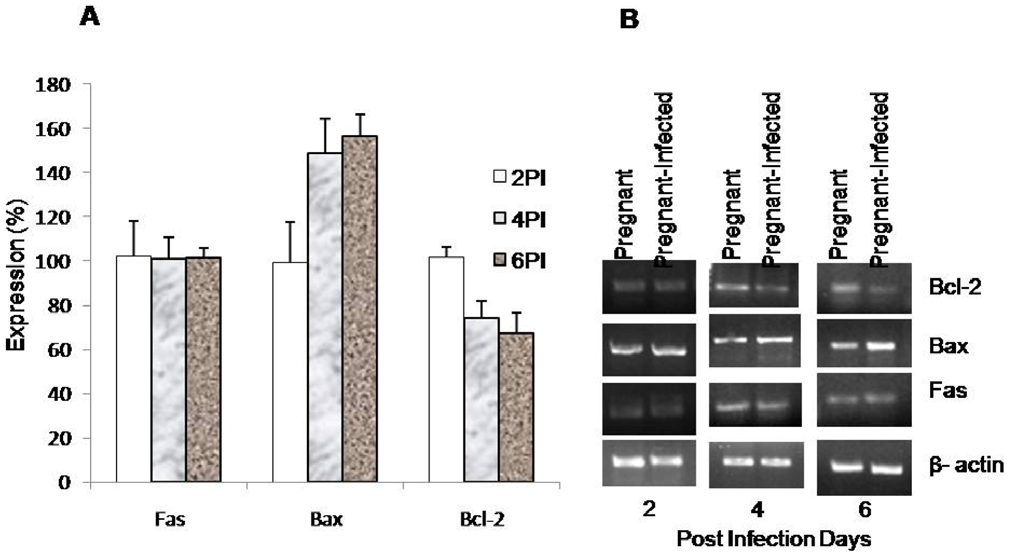
Up regulation of Bax and down regulation of Bcl-2 expression in placenta of *P. berghei* infected mice. The expression of Fas, Bax and Bcl-2 in placenta of both pregnant and pregnant-infected mice was studied by RT-PCR. β-actin was used as internal (positive) control. (A): The bar diagram represents the densitometric alalysis of the Fas, Bax and Bcl-2 expression (percent relative to non-infected where non-infected was considered as 100%). (B): The gel photograph is representative of 3 separate experiments.

Furthermore, the expression of Fas, Bcl-2 and Bax, the apoptosis markers were monitored by RT-PCR in the placenta. It was found that the Fas expression did not alter much in *P.berghei* infected placenta compared with that in normal placenta at each point of observation. However, the results showed the up-regulation of Bax and down-regulation of Bcl-2 proteins with the progression of malarial infection (Figure 9).

### Increased activity of caspase 3 and Caspase 9

**Figure 10:**
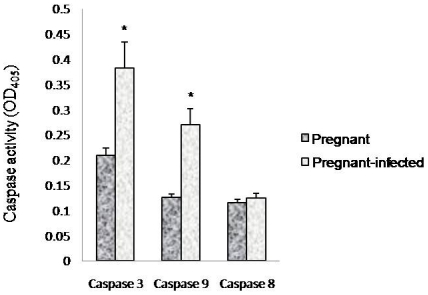
Increased activity of caspase 3 and 9 and decreased caspase 8 activity in placenta of *P. berghei* infected mice. Activities of caspases were measured in placenta of pregnant and pregnant-infected mice on day 6 PI. Values are expressed as mean ± standard deviation. ^*^(p<0.05) vs. pregnant.

The occurrence of apoptosis in malaria infected placenta was further confirmed by measuring the activities of caspase 3, caspase 8 and Caspase 9. We found increased activity of caspase 3 and Caspase 9 in infected placenta, while no change in the activity of caspase 8 was found (Figure 10).

## Discussion

Oxidative stress has been implicated in pathogenesis of many diseases including malaria [25], [26], [27]. During malarial infection, the antigenic stimulation activates the immune system of the body thereby causing release of reactive oxygen species (ROS) [28], [29]. In addition to host's immune system, malaria parasite also produces ROS resulting into haemoglobin degradation [30], [31]. However, oxidative stress during malarial infection is beneficial to the patient to combat the intra erythrocytic parasite. Excessive production of ROS by recruited monocytes and neutrophyls at vascular lining, damage the cells and tissues [32], [33], [34]. In the present study, high levels of MDA, the index of oxidative stress have been found in the placenta of *P .berghei* infected mice, which correlated well with increase in percent parasitaemia with progression of infection. This increase in MDA level may be due to the inefficient antioxidant defence system, which is evident by decrease in the activity of catalase and unaltered levels of SOD and GSH. Kulkarni et al. (2003) have also found the decreased levels of antioxidants during malarial infection to be responsible for increased oxidative stress [35]. However, Pabon et al. (2003) have observed that high oxidative stress during malarial infection is due to increased lipid peroxidation rather than from reduced antioxidant [36]. In addition, high levels of MDA in malaria infected placenta may also be due to the ROS production by infilterated immune cells and infected erythrocytes in placental sinuses which may cause trophoblast cells damage. In cerebral malaria too ROS production by massively recruited and activated monocytes and neutrophils at blood brain barrier has been found to be responsible for endothelial cell lining damage [37], [38]. Moreover, increased concentrations of TNF-α, IFN-γ and IL-2 in placenta during malarial infection have also been reported to be associated with poor outcomes in human pregnancies. However, in multigravidae high concentrations of IFN-γ in placenta are responsible for protection against malaria [39]. It seems that recruitment of immune cell in inter-villous spaces of placenta and associated increased TNF-α concentration and oxidative stress are responsible for placental pathology and pregnancy outcome during malarial infection.

Furthermore, to investigate whether oxidative stress induces apoptosis in placenta, apoptosis markers, such as DNA fragmentation, ethedium bromide/acridine orange staining, expression of Fas, Bcl-2 ans Bax proteins by RT-PCR and activation of caspases were studied. DNA fragmentation assay, ethedium bromide/acridine orange staining and activation of caspase 3 clearly demonstrated the occurrence of apoptosis in placenta that begin after day 2 PI. It is known that apoptosis involve two pathways, either death receptor pathway (Fas mediated) or mitochondrial pathway [40], [41] whereas mitochondrial pathway of apoptosis is mainly initiated by down-regulation of antiapoptotic protein e.g., Bcl-2 and Bcl_x_L and up-regulation of pro-apoptotic proteins e.g., Bax, Bak and Bid. Pro-apoptotic proteins induce apoptosis by forming pores in mitochondria, releasing cytochrom-c to the cytosol and binding to anti-apoptotic proteins to antagonizing their action [42], [43]. In the present study, the apoptosis occurring in malaria infected placenta did not involve death receptor pathway as no change in the expression of Fas gene and activation of caspase 8 was observed compared with non-infected placenta. However, the up-regulation of Bax expression and down-regulation of Bcl-2 in placenta of *P. berghei* infected seems to be responsible for inducing mitochondrial pathway of apoptosis. Moreover, the activation of caspase 9 in malaria infected placenta also indicated the occurrence of mitochondrial pathway of apoptosis. In the present study, increased ROS production, oxidative stress and mitochondrial pathway of apoptosis in malaria infected placenta are in accordance with earlier studies, where it has been documented that the generation of ROS and associated oxidative stress are involved in mitochondrial pathway of apoptosis [44], [45]. However, the present finding is contrary to the Crocker et al. (2004) study, where no change in apoptosis has been found in placental cells during malarial infection [14]. This difference may be due the different parasite strain and host. Moreover, the time when placental pathology was studied is also different in both studies. In our study placental pathology was studied on GD 16 that is start of 3rd trimester while Crocker et al. (2004) have studied placenta pathology in women after delivery.

Based on the present observation it can be proposed that malaria infection in the placenta augments oxidative stress, which in turn activate mitochondrial pathway of apoptosis leading to placental damage. Thereby, suggesting that all these alterations occurring at the materno-fetal exchange system may result into placental insufficiency leading to intra-uterine growth retardation and low birth weight.
